# Coastline Detection with Gaofen-3 SAR Images Using an Improved FCM Method

**DOI:** 10.3390/s18061898

**Published:** 2018-06-11

**Authors:** Meng An, Qian Sun, Jun Hu, Yuqi Tang, Ziwei Zhu

**Affiliations:** 1 School of Geosciences and Info-physics, Central South University, Changsha 410083, China; AnMeng@csu.edu.cn (M.A.); csuhujun@csu.edu.cn (J.H.); yqtang@csu.edu.cn (Y.T.); zhumyrtle@csu.edu.cn (Z.Z.); 2 College of Resources and Environmental Science, Hunan Normal University, Changsha 410081, China; 3 Key Laboratory of Geospatial Big Data Mining and Application, Hunan Normal University, Changsha 410081, China; 4 Key Laboratory of Metallogenic Prediction of Nonferrous Metals and Geological Environment Monitoring (Central South University), Ministry of Education, Changsha 410083, China

**Keywords:** Gaofen-3, SAR, coastline detection, Bohai, Taihu, FCM, wavelet decomposition

## Abstract

The coastline detection is one of the main applications of the Gaofen-3 satellite in the ocean field. However, the capability of Gaofen-3 SAR image in coastline detection has not yet been validated. In this paper, two Gaofen-3 SAR images, acquired in 2016, were used to extract the coastlines of the regions of Bohai and Taihu in China, respectively. The classical Fuzzy C-means (FCM) method was used in the coastline detection, but had been improved by combining the Wavelet decomposition algorithm to better suppress the inherent speckle noises of SAR image. Coastline detection results obtained from two Sentinel-1 SAR images acquired on the same regions were compared with those of the Gaofen-3 images. By using the manually delineated coastlines as the standards in the qualitative evaluations, improvements of about 12.0%, 8.3%, 23.8%, and 9.4% can be achieved by the improved FCM method with respect to the indicators of mean, RMSE, PGSD, and P90%, respectively; demonstrating that the Gaofen-3 data is superior to the Sentinel-1 data in the detection of coastline.

## 1. Introduction

A coastline is the continuous boundary between land and ocean masses. It is the baseline for dividing the administrative area of the ocean and the land, and serves as the dividing line between the ocean depth datum and the land elevation datum. Monitoring the dynamic change of coastline is of great significance for the ocean management [1,2,3,4].

Field measurements (e.g., GPS and total station) are the traditional methods for coastline detection. Although the field detection of the coastline is detailed and accurate, it is time-consuming and laborious, and thus difficult to conduct in large area [4]. Optical remote sensing provides a fast and effective method for detecting dynamic changes of coastline [5]. However, its application are greatly limited by weather and tide conditions. Recently, coastline detection from synthetic aperture radar (SAR) image has had faster growing development due to its wide spatial coverage, high resolution, strong penetration ability, and all-day/all-weather imaging capabilities [6].

In recent years, many scholars have studied the automatic coastline detection technologies with SAR images. Modava et al. [2] combined the active contour model and spatial fuzzy clustering to test the ALOS-2 ScanSAR images in Strait of Glibraltar and a part of the Alboran Sea. Horritt [7] segmented the ERS-1 SAR image of the UK’s east coast by statistical active contour model on homogeneous speckle statistics region. Liu et al. [8] combined the region-based and edge-based active contour models in two different scales, and tested the algorithm with TerraSAR-X and RADARSAT-2 date. Hao et al. [9] extracted the RADARSAT image gradient in a coastal area by using Roberts operator, and then used Maximum Entropy to segment SAR image. Zhang et al. [10] adopted mathematical morphology and the Otsu algorithm to extract coastline in ENVISAT ASAR images. Xie [11,12] employed two RADARSAT ScanSAR images of multiple ships and multiple islands for costaline detection based on the seeds growing method. Niedermeier et al. [13] applied an edge detection method to determine the boundary by a blocktracing algorithm, and used an active contour model algorithm to detect coastline in the Elbe estuary with the ERS-2 SAR image. Alonso et al. [14] presented an unsupervised edge enhancement algorithm based on a combination of wavelet coefficients at different scales with RADARSAT-1, ENVISAT, and ERS-1 images, respectively. Zhang [15] applied stationary wavelets to decompose ENVISAT image over the eastern coast of the mainland of China, and obtained the coastline according to wavelet coefficient gradient information. Ding et al. [16,17,18] achieved the extraction of water-land boundaries from ENVISAT and COSMO-SkyMed SAR data, respectively. Nunziata et al. [19,20] exploited the COSMO-SkyMed SAR data to distinguish sea and land in the North Sea and Gulf of Naples. Gou et al. [21] extracted polarimetric and texture information from the three channels of ALOS-2 PALSAR images to distinguish sea and land. Wang et al. [22] proposed a classification chain for detecting sediments in intertidal zones with ALOS PALSAR-2 data. Buono et al. [23] tested the F–D and the H/α classification algorithms with RADARSAT-2 SAR images in the Yellow River delta.

The Gaofen-3 satellite was launched by the China Academy of Space Technology (CAST) on 10 August 2016, which is the first multi-polarization C-band SAR imaging satellite in China with a resolution of up to 1 m [24], also the only civil microwave remote sensing satellite in the “National high resolution to earth observation system major project” [25]. The Gaofen-3 satellite has the characteristics of high resolution, large imaging width, high radiation precision, and multi-imaging mode. It can monitor the global ocean and land in all day and all day, and expand the range of observation and promote the quick response ability through the left and right attitude maneuvers [25]. The Gaofen-3 satellite can switch between 12 specific modes according to the bandwidth and spatial resolution requirements, and freely switch the spatial resolution in a range from 500 to 1 m. Meanwhile, the Gaofen-3 satellite has double-polarizations and full-polarizations, which can greatly expand the observation and application ability [26]. Main technical specifications of Gaofen-3 satellite are shown in Table 1. 

The successful launch of the Gaofen-3 satellite has changed the status of China’s civilian high resolution SAR images relying on imports, which can provide high-quality, high-precision earth observation SAR data for users in both domestic and overseas [25]. In this paper, we focus on demonstrating the applicability of the Gaofen-3 images in the detecting of coastlines by carrying out the experiments in Bohai and Taihu, China, respectively. In order to reduce the noise interferences in the Gaofen-3 images, the classical Fuzzy C-means (FCM) algorithm is employed due to its good clustering effect and low computational complexity, but has been improved by integrating the wavelet decomposition algorithm to better suppress the inherent speckle noises of SAR image. In addition, comparisons are conducted between the Gaofen-3 data and the Sentinel-1 data acquired over the same region. To our knowledge, the results demonstrate the first attempt for coastline detection with Gaofen-3 data. The remainder of this paper is organized as follows. Section 2 introduces the Gaofen-3 SAR data and the test sites used. Detail algorithms for the coastline detection are presented in Section 3. Then, the results of Gaofen-3 data and the assessment are given in Section 4. Conclusions are summarized in Section 5.

## 2. Experimental Sites and Used Data

### 2.1. Experimental Sites

**Figure 1:**
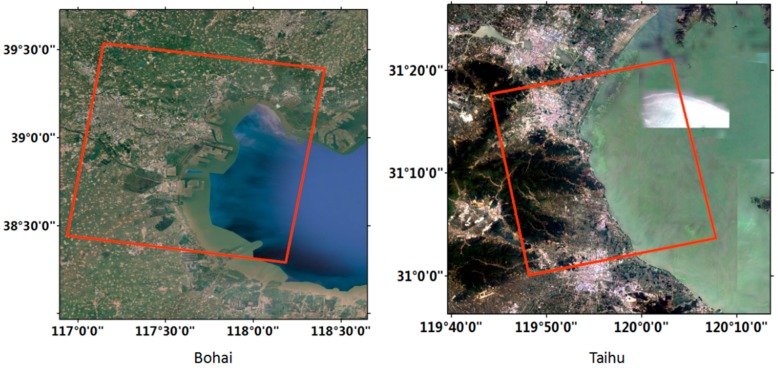
Experimental sites and their specific locations in China. Red box indicates the coverage of the used Sentinel-1 data.

In this study, experimental data was selected from the test sites in Bohai, Tianjin Province and Taihu, Anhui Province. The specific locations are shown in Figure 1.

Bohai is surrounded by the northern Liaodong Bay, the western Bohai Bay, the southern Laizhou Bay, central basin, and the Bohai Strait [27]. Bohai Bay is a semi-enclosed sea located at the north of China [28]. The seabed terrain is from the shore in the south to the sea lean in the north. There are large amounts of sediments in coastal rivers, which are mainly constituted by fine particles of silt and sludge. Its western coast is Tianjin Port, which is densely populated and has industrialized zones, with rich oil and gas resources, and is the fifth largest port in the world [29]. Since Bohai Bay is an important economic development zone, there are many ports zone and many kinds of aquaculture farms in the coastal [30]. Moreover, there are many rivers and long shallow sea beaches in the area, forming a good wetland landscape.

Taihu is located in the southern margin of Changjiang delta [31], which is one of the five largest freshwater lakes in China. The Taihu basin is dominated by the plain, the terrain is characterized by high surrounding and low middle, forming a natural basin. The middle of Taihu is a plain and low-lying land, and Tianmu Mountain, the Maoshan and the foothills are located in the West. Vegetation is mainly distributed in hilly and mountainous areas of the Taihu basin. In addition, there is a certain amount of cyanobacteria in the water.

### 2.2. Used Data

**Figure 2:**
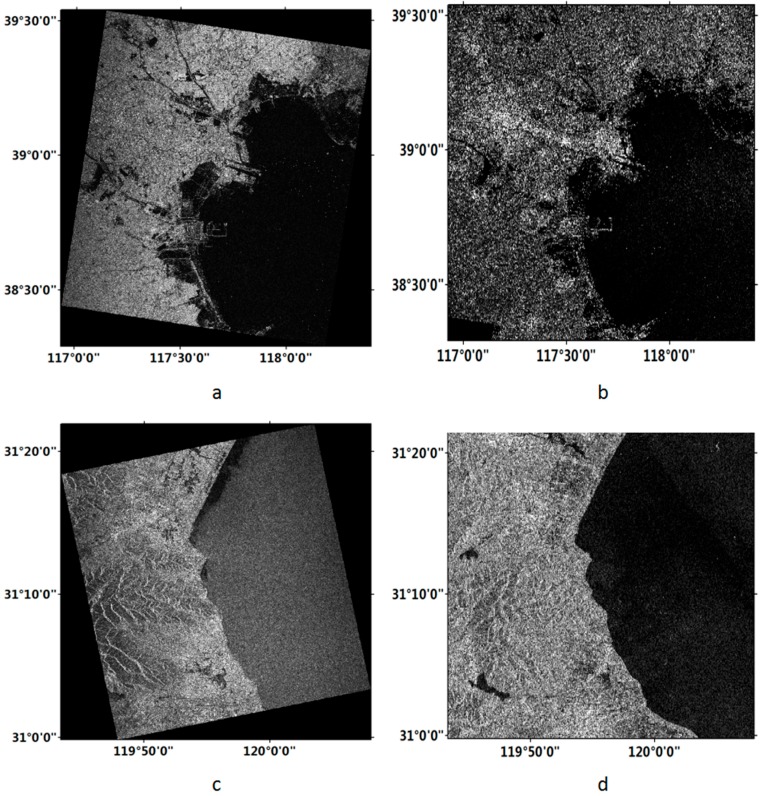
Used images of the testing sites. (**a**) Gaofen-3 image of Bohai; (**b**) Sentinel-1 image of Bohai; (**c**) Gaofen-3 image of Taihu; (**d**) Sentinel-1 image of Taihu.

In this study, we used the Gaofen-3 satellite images in the regions of the Bohai Bay and the Western of Taihu, both of which were acquired on 20 August 2016. The Gaofen-3 images are shown in Figure 2a,c. The detailed information of the Gaofen-3 data is shown in Table 2.

In order to verify the performance of Gaofen-3 data in the coastline detection, the Sentinel-1 data, which were provided by the SAR satellite launched by ESA in 2014, were acquired over the same regions in this study. It should be highlighted that the Sentinel-1 data is the unique source of publicly downloadable data in the world. Since it is quite difficult to obtain the Sentinel-1 images acquired at the same date as the Gaofen-3 data in the study areas, we choose the Sentinel-1 data acquired at the closest time. The Sentinel-1 images are shown in Figure 2b,d. The basic information of the Sentinel-1 data is also shown in Table 2.

## 3. Method

In this study, the classical FCM algorithm was improved for the coastline detection with Gaofen-3 image by combining it with stationary wavelet decomposition.

FCM is an unsupervised clustering algorithm initially, which has a low computational complexity [32]. The main idea of this method is distributing the data into clusters that minimizes the similarity degree among the objects divided into the same cluster, and maximizes the dissimilarity among the different clusters [33]. The FCM cost function can be defined as follows [34]:

(1) $$J\left(U,c_{1},\ldots c_{c}\right)=\sum_{i=1}^{c}J_{i}=\sum_{i=1}^{c}\sum_{j}^{n}\mu_{ij}^{m}d_{ij}^{2}=\sum_{i=1}^{c}\sum_{j}^{n}\mu_{ij}^{m}\left\|c_{i}-x_{j}\right\|$$

where *μ* is the membership of pixel *x_j_* in the *i*th cluster, which ranges from 0 to 1. *c_i_* is the cluster center of the *i*th cluster. *m* represents a weighted index greater than 1, which controls the fuzziness of the segmentation results.

The membership function indicates the degree of a pixel belonging to a fuzzy set with the value between 0 and 1 [35]. Its size depends on the distance to the cluster center, and the sum of the membership value of a pixel at all cluster centers is 1. The pixels close to the cluster center have a high membership values, and low membership values are allocated to the pixels far from the cluster center [34]. The membership function and cluster center are updated as below [34]:

(2) $$\mu_{\mathrm{ij}}=\frac{1}{\sum_{k=1}^{c}{\left(\frac{\left\|x_{j}-c_{i}\right\|}{\left\|x_{j}-c_{k}\right\|}\right)}^{2/(m-1)}}$$

(3) $$c_{i}=\frac{\sum_{j=1}^{N}\mu_{ij}^{m}x_{j}}{\sum_{j=1}^{N}\mu_{ij}^{m}}$$

However, using SAR images in coastline detection is a difficult task due to the inherent speckle noise. Most denoising methods are based on spatial domain, such as Lee [36], Frost [37], and Kuan [38] filters and their enhanced versions [39]. These algorithms can work well in noise reduction, but might destroy feature edge due to the reduction of the spatial resolution. In addition, wavelet-based denoising methods has been studied in SAR images [40,41]. Since the speckle noise exists in the high frequency region, the wavelet transform with good time-frequency characteristics has great potential to reduce the speckle noise as well as to preserve feature edge [42], which can be assessed by employing the smoothness index (SI) and edge preserved index (ESI) [43,44,45]. Therefore, in this paper we employed the wavelet decomposition to obtain the preliminary denoising SAR image, which will be used in the process of the FCM algorithm. In order to make the sub-band images have the same size as the original one, the stationary wavelet decomposition method was used [15]. First, we decomposed the original SAR image into four sub-band images corresponding to approximation, vertical, horizontal, and diagonal coefficients. Since the approximation, vertical, and horizontal sub-band images all include low-pass signals, we then calculated the mean of the sub-band images, except for the diagonal one that only included high-pass signals, to generate the preliminary denoising SAR image. Although the vertical and horizontal sub-band images also included high-pass signals, they were used to preserve the edge information that also behaved as high-pass signals.

**Figure 3:**
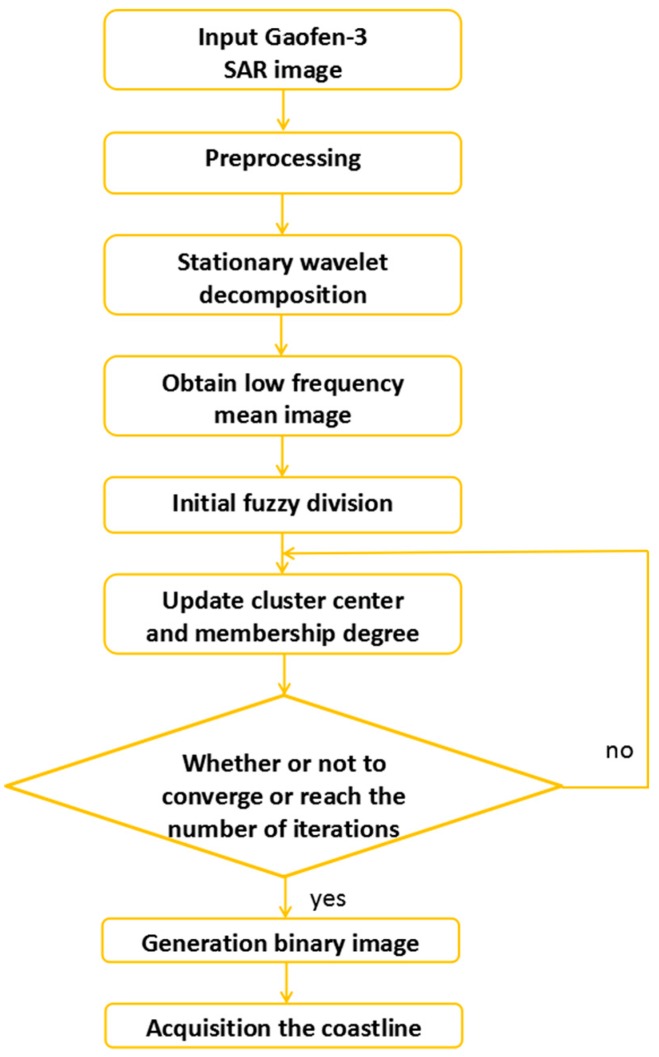
Flow chart of the improved Fuzzy C-means (FCM) algorithm.

Subsequently, we needed to dispose the experimental image by the FCM clustering method. First, we initialized the cluster center and the related parameters, such as the number of classifications and the weighting exponents. According to the initial conditions and Equation (3), a new cluster center was obtained. Then we used Equation (2) to calculate the distance from each pixel to the new cluster center. This was an iterative calculation until iterations converged or reached a set number. The flow chart of the improved FCM algorithm is shown in Figure 3.

## 4. Results and Analysis

### 4.1. Accuracy Evaluation Method

The accuracy evaluation method proposed in reference [46] was used in this study. Field surveying is not only time-consuming and laborious, but also inapplicable in some special areas. Therefore, the delineated manually coastline is considered as an actually coastline, which can be used as a standard for accuracy assessment. In this study, we employed the Digital Shoreline Analysis System (DSAS) tool [47,48] to calculate the change between the delineated and the automatic detected coastlines by using the proposed method. The DSAS is a software extension of ArcGIS for calculating coastline change. More details can be found in references [47,48]. In addition, the baseline is required when the accuracy is evaluated [49]. 

First, the test coastline and the image were imported into the DSAS software, and we delineated the coastline manually. Then, a baseline was retrieved from a buffer built based on the extracted coastlines by removing the redundant parts. The baseline was the datum line to determine the directions of the transact lines, since the true and detected coastlines had different orientations [50]. Then, we generated transact lines which were perpendicular to the baseline. The transact lines intersected with the true and detected coastlines. Finally, based on the distance between the intersection points in true and detected coastlines we calculated the error with respect to the selected indexes. There are four indexes used to evaluate the accuracy, including the mean, RMSE, P90%, and PGSD [46]. The implications of P90% and PGSD were interpreted as follows:

P90%: 90% of the sampling points error within the distance.

PGSD: The percentage of sampling points within one pixel distance of the total sampling points.

**Figure 4:**
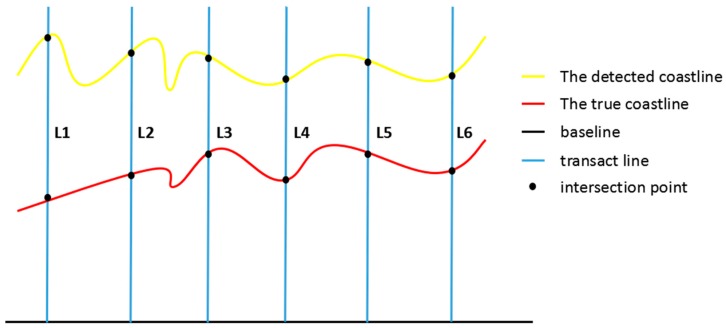
Principle of accuracy evaluation.

The principle of accuracy evaluation is shown in Figure 4. In this paper, we selected a point every 200 m for accuracy evaluation. It should be noted that the smaller the indexes, the better the results, except the PGSD.

### 4.2. Experimental Results in Bohai Region

**Figure 5:**
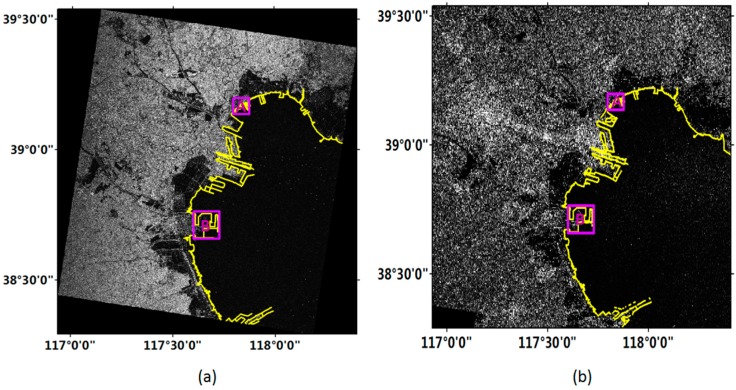
Coastline detection results from the improved FCM method in Bohai region. (**a**) Gaofen-3 image; (**b**) Sentinel-1 image. The boxes indicate the locations of the selected sub-regions.

Figure 5a,b shows the coastline detection results from the improved FCM method in the Bohai region with Gaofen-3 and Sentinel-1 images, respectively. In order to provide quantitative assessment, two sub-regions of the image were selected to carry out the accuracy evaluation, as indicated by the boxes in Figure 5. One was an artificial coast with a clear sea and land contrast, and the other was containing an aquaculture area.

**Figure 6:**
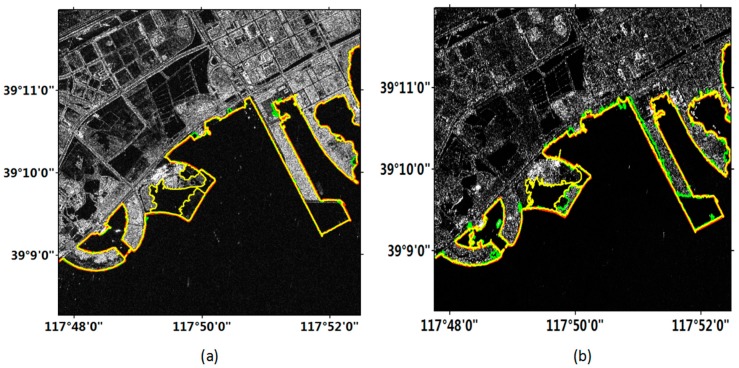
Experimental results of sub-region A in Figure 5. Red line represents the manually delineated coastline. Yellow and green lines represent the coastline detection results from the improved and original FCM methods, respectively. (**a**) Gaofen-3 image; (**b**) Sentinel-1 image.

Figure 6 exhibits the coastline detection results in the sub-region A labeled in Figure 5. This area was mainly artificial coast without complex building, and the contrast between sea and land was obvious. In Figure 6, we can see that the quality of the Gaofen-3 image was very high, and the objects were clear. Sentinel-1 image was basically correct in displaying the objects, but in some areas the boundary between the land and the sea was obscured due to the low intensity of the land. Moreover, the coastline detected in the Gaofen-3 image was relatively smooth, but the Sentinel-1 image was rough and contained many branches. Although the coastlines detected by the two methods (i.e., the improved and original FCM methods) in the Gaofen-3 image were generally consistent with the manually delineated coastline, there were some errors with the results of the original FCM method. 

Quantitative evaluations can be found in Table 3. It was shown that in the original FCM method detection results, the Sentinel-1 image was superior to the Gaofen-3 image. However, by using the improved FCM method, the results of the Gaofen-3 image were better than those of the Sentinel-1 images with respect to the four indexes as a whole, although the RMSE of the Gaofen-3 image was a little larger than that of the Sentinel-1 image. In addition, the results of the improved FCM method were superior to those of the FCM method. 

**Figure 7:**
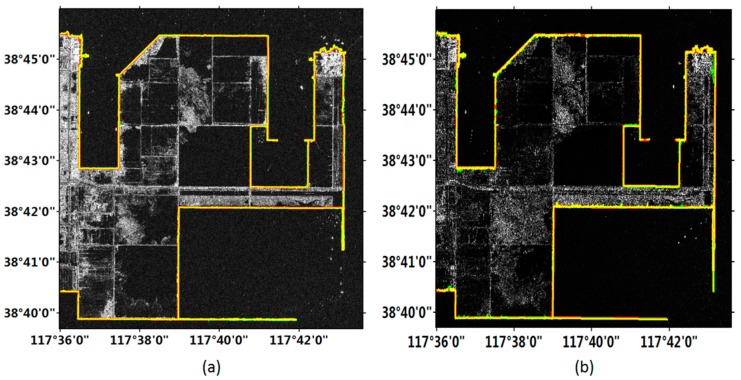
Experimental results of sub-region B in Figure 5. Red line represents the manually delineated coastline. Yellow and green lines represent the coastline detection results from the improved and original FCM methods, respectively. (**a**) Gaofen-3 image; (**b**) Sentinel-1 image.

**Figure 8:**
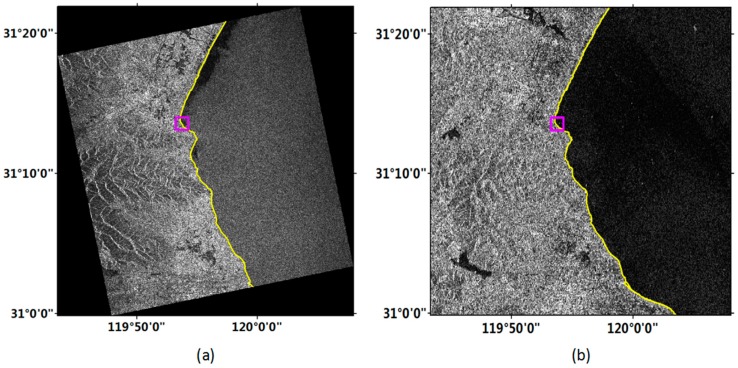
Coastline detection results from the improved FCM method in Taihu region. (**a**) Gaofen-3 image; (**b**) Sentinel-1 image. The box indicates the location of sub-region.

Figure 7 exhibits the coastline detection results in the sub-region B labeled in Figure 5. This area included a distinct artificial coast and a mariculture with fences. It is observed in Figure 8 that the image obtained by the Gaofen-3 satellite clearly show the land. The coastline detected in the Gaofen-3 image was smooth. Although the fence was displayed as a straight line in the image, it could be detected by using the both methods. Similarly, the Sentinel-1 image also clearly showed the objects, but in several areas the coastline was discontinuous.

Quantitative evaluations are shown in Table 4. We can see that the results of the Gaofen-3 image with the original and improved FCM methods were both superior to those of the Sentinel-1 image with respect to the four indexes. And again, the improved FCM method had been proved to be superior to the original FCM method for detecting the coastline.

### 4.3. Experimental Results in Taihu Region

Figure 8a,b shows the coastline detection results from the improved FCM method in Taihu region with Gaofen-3 and Sentinel-1 images, respectively. Since this area was relatively unitary and had no complex artificial buildings, one sub-region of the image were selected to carry out the accuracy evaluation, as indicated by the box in Figure 8.

**Figure 9:**
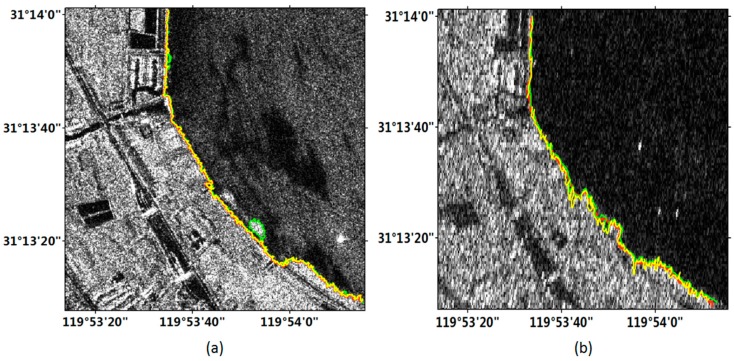
Experimental results of sub-region in Figure 8. Red line represents the manually delineated coastline. Yellow and green lines represent the coastline detection results from the improved and original FCM methods, respectively. (**a**) Gaofen-3 image; (**b**) Sentinel-1 image.

Figure 9 exhibits the coastline detection results in the sub-region labeled in Figure 8. This area belonged to an inland lake, there are few complex artificial buildings, and the land was relatively simple. From Figure 9, we can see that the Gaofen-3 image clearly showed the landscape but with some noises. Since the backscattering coefficient of water vegetation on radar image was larger than that of waters, water vegetation will also be shown on the image. The coastlines detected by the two methods in the Gaofen-3 image were similar with the manually delineated coastline in the whole, although there are little errors in the local regions. The Sentinel-1 image also correctly displayed the local objects, and the detection results of the improved FCM method were conformity with the manually delineated coastline.

Quantitative evaluations can be found in Table 5. We can see that the Gaofen-3 image detection results with the original and improved FCM methods were both superior to those of Sentinel-1 image detection results with respect to the four indexes. However, the PGSD index of Gaofen-3 image detection results was worse than that of Sentinel-1 image detection results in original FCM method. This could be ascribed to the existence of vegetation in water, which degraded the detection precision in the disturbed area. As expected, in Taihu region the improved FCM method was also superior to the original FCM method for detecting the coastline with respect to the four indexes. 

## 5. Conclusions

In this paper, the performance of the Gaofen-3 SAR data on the coastline detection was examined over the Bohai and Taihu regions in China. A novel method, which combined the classical FCM method and wavelet decomposition algorithm, was proposed for the coastline detection with SAR image. The results showed that the improved FCM method can better suppress the noise interferences in the Gaofen-3 images than the original FCM method. More importantly, it was found that the quality of the Gaofen-3 image was generally better than that of the Sentinel-1 image, which is greatly beneficial for the extraction of the details of coastline. The quantitative assessments were also conducted by comparing the detected coastlines with the manually delineated coastline. It was found that the Gaofen-3 data was superior to the Sentinel-1 data in the coastline detection, yielding improvement of about 12.0%, 8.3%, 23.8%, and 9.4% with respect to the indicators of the mean, RMSE, PGSD, and P90%, respectively. The results demonstrate that the Gaofen-3 image was more suitable for the detection of different kinds of coastlines than the Sentinel-1 image, and can meet the basic requirements of national geo-survey. However, the detection precisions of Gaofen-3 image were vulnerable to the turbid sea environment, where the boundary between sea and land was not obvious. Future research can improve the results of this study by employing the state-of-art speckle noise filters. 

## Figures and Tables

**Table 1:** Main technical specifications of Gaofen-3 satellite.

Imaging Modes		Resolution (m)			Imaging Bandwidth (km)		Incidence Angle (°)	Polarization Mode
		Nominal	Azimuth	Range	Nominal	Size		
spotlight		1	1.0~1.5	0.9~2.5	10 × 10	10 × 10	20~50	Single
ultra-fine strip		3	3	2.5~5	30	30	20~50	Single
fine strip I		5	5	4~6	50	50	19~50	Dual
fine strip II		10	10	8~12	100	95~110	19~50	Dual
standard strip		25	25	15~30	130	95~150	17~50	Dual
narrow scan		50	50~60	30~60	300	300	17~50	Dual
wide scan		100	100	50~110	500	500	17~50	Dual
global		500	500	350~700	650	650	17~53	Dual
full polarized Strip I		8	8	6~9	30	20~35	20~41	Full
full polarized Strip II		25	25	15~30	40	35~50	20~38	Full
wave imaging		500	500	350~700	650	650	20~41	Full
extended	low	25	25	15~30	130	120~150	10~20	Dual
	high	25	25	20~30	80	70~90	50~60	Dual

**Table 2:** Basic information of the used data.

Satellites	Areas	Imaging Modes	Orbits	Acquired Dates	Range Spacing	Azimuth Spacing
Gaofen-3	Bohai	FSⅡ	Descending	20 August 2016	10.0 m	10.0 m
	Taihu	UFS	Ascending	20 August 2016	3.0 m	3.0 m
Sentinel-1	Bohai	ISP	Descending	3 November 2016	2.3 m	14.0 m
	Taihu	ISP	Descending	19 August 2016	2.3 m	14.0 m

**Table 3:** Quantitative evaluation in the sub-region A of Bohai.

Method	Data Type	Mean (m)	RMSE (m)	P90% (m)	PGSD (%)
Improved FCM	Gaofen-3	5.77	5.89	10.07	94.37
	Sentinel-1	6.30	5.83	14.03	80.00
Original FCM	Gaofen-3	6.97	7.66	13.87	90.70
	Sentinel-1	8.53	4.81	13.14	90.00

**Table 4:** Quantitative evaluation in the sub-region B of Bohai.

Method	Data Type	Mean (m)	RMSE (m)	P90% (m)	PGSD (%)
Improved FCM	Gaofen-3	4.09	4.85	8.05	94.47
	Sentinel-1	5.29	5.07	11.81	94.04
Original FCM	Gaofen-3	10.39	7.63	18.31	80.88
	Sentinel-1	11.46	7.70	20.85	72.02

**Table 5:** Quantitative evaluation in the sub-region of Taihu.

Method	Data Type	Mean (m)	RMSE (m)	P90% (m)	PGSD (%)
Improved FCM	Gaofen-3	4.32	3.35	10.76	80.00
	Sentinel-1	4.55	4.27	12.12	72.88
Original FCM	Gaofen-3	5.80	7.64	15.08	65.38
	Sentinel-1	6.34	10.40	15.90	68.33

## References

[1] Liu H., Jezek K.C. (2004). Automated Extraction of Coastline From Satellite Imagery by Integrating Canny Edge Detection and Locally Adaptive Thresholding Methods. Int. J. Remote Sens..

[2] Modava M., Akbarizadeh G. (2017). Coastline Extraction from SAR Images Using Spatial Fuzzy Clustering and the Active Contour Method. Int. J. Remote Sens..

[3] Liu B.Y., Su F.Z. (2005). Remote Sensing Investigations on Coast Zones and Islands in China: Principles, Methods and Systems.

[4] Shen J.S., Zhai J.S., Guo H.T. (2009). Study on Coastline Extraction Technology. Hydrogr. Surv. Charting.

[5] Zhang M., Jiang X.Z., Zhang J.R., Tian B. (2008). Developments of Coastline Detection with Remote Sensing Data. Yellow River.

[6] Ding X.W., Li X.F. Coastline Detection in SAR Images Using Multiscale Normalized Cut Segmentation. Proceedings of the IEEE International Geoscience and Remote Sensing Symposium.

[7] Horritt M. (1999). A statistical active contour model for SAR image segmentation. Image Vis. Comput..

[8] Liu C., Xiao Y.Y., Yang J. (2017). A Coastline Detection Method in Polarimetric SAR Images Mixing the Region-Based and Edge-Based Active Contour Models. IEEE Trans. Geosci. Remote Sens..

[9] Jing H., Chen X.Q., Gu Z.W. (2006). A Method for Coastline Extraction Based on Edges. Comput. Simul..

[10] Zhang L., Chen H.H. (2011). Coastline detection in SAR imagery based on Otsu algorithm and mathematical morphology. Microcomput. Inf..

[11] Xie M.H., Zhang Y.F., Fu K. (2006). Coastline Detection Algorithm of SAR Image Based on Seeds Growing. Comput. Dev. Appl..

[12] Xie M.H., Zhang Y.F., Fu K. (2007). Algorithm of Detection Coastline from SAR Images Based on Seeds Growing. J. Grad. Sch. Chin. Acad. Sci..

[13] Niedermeier A., Romaneeßen E., Lehner S. (2000). Detection of Coastlines in SAR Images Using Wavelet Methods. IEEE Trans. Geosci. Remote Sens..

[14] Alonso M.T., Lopez-Martinez C., Mallorqui J.J., Salembier P. (2010). Edge Enhancement Algorithm Based on the Wavelet Transform for Automatic Edge Detection in SAR Images. IEEE Trans. Geosci. Remote Sens..

[15] Zhang P., Li H.P. (2010). Sea Boundary Extraction in SAR Satellite Images based on Stationary Wavelet Transform. Comput. Dev. Appl..

[16] Ding X.W., Li X.F. (2011). Monitoring of the Water-area Variations of Lake Dongting in China with ENVISAT ASAR images. Int. J. Appl. Earth Obs. Geoinf..

[17] Ding X.W., Li X.F. (2014). Shoreline Movement Monitoring based on SAR images in Shanghai, China. Int. J. Remote Sens..

[18] Ding X.W., Nunziata F., Li X.F., Migliaccio M. (2017). Performance Analysis and Validation of Waterline Extraction Approaches Using Single- and Dual-Polarimetric SAR Data. IEEE J. Sel. Top. Appl. Earth Obs. Remote Sens..

[19] Nunziata F., Migliaccio M., Li X. Dual-polarized COSMO-SkyMed SAR data for Coastline Detection. Proceedings of the IEEE International Geoscience and Remote Sensing Symposium.

[20] Nunziata F., Migliaccio M., Li X., Ding X. (2013). Coastline Extraction Using Dual-Polarimetric COSMO-SkyMed PingPong Mode SAR Data. IEEE Geosci. Remote Sens. Lett..

[21] Gou S., Li X., Yang X. (2016). Coastal Zone Classification with Fully Polarimetric SAR Imagery. IEEE Geosci. Remote Sens. Lett..

[22] Wang W.S., Yang X.F., Li X.F., Chen K.S., Liu G.H., Li Z.W., Gade M. (2017). A Fully Polarimetric SAR Imagery Classification Scheme for Mud and Sand Flats in Intertidal Zones. IEEE Trans. Geosci. Remote Sens..

[23] Buono A., Nunziata F., Migliaccio M., Yang X.F., Li X.F. (2017). Classification of the Yellow River Delta Area Using Fully Polarimetric SAR Measurements. Int. J. Remote Sens..

[24] Shao W.Z., Sheng Y.X., Sun J. (2017). Preliminary Assessment of Wind and Wave Retrieval from Chinese Gaofen-3 SAR Imagery. Sensors.

[25] Zhang Q.J. (2017). System Design and Key Technologies of the GF-3 Satellite. Acta Geod. Cartogr. Sin..

[26] Wang T.Y., Zahng G., Yu L., Zhao R.S., Deng M.J., Xu K. (2017). Multi-Mode GF-3 Satellite Image Geometric Accuracy Verification Using the RPC Model. Sensors.

[27] Yang J.Q., Luo X.X., Shi H.H., Leng Y. Chlorophyll-a Spatial Distribution in Bohai Sea from 2006 to 2007 I-Six Important Regions. Proceedings of the International Conference on Challenges in Environmental Science and Computer Engineering.

[28] Zou S.C., Xu W.H., Zhang R.J., Tang J.H., Chen Y.J., Zhang G. (2011). Occurrence and Distribution of Antibiotics in Coastal Water of the Bohai Bay, China: Impacts of River Discharge and Aquaculture Activities. Environ. Pollut..

[29] Gao X.L., Chen C.T. (2012). Heavy Metal Pollution Status in Surface Sediments of the Coastal Bohai Bay. Water Res..

[30] Zheng G.X., Bai J., Zhang S. Bohai Bay Sea Ice Monitoring Based on the HJ Satellite Images. Proceedings of the 7th International Congress on International Congress on Image and Signal Processing.

[31] Qin B.Q., Xu P.Z., Wu Q.L., Luo L.C., Zhang Y.L. (2007). Environmental Issues of Lake Taihu, China. Hydrobiologia.

[32] Bezdek J.C. (1981). Pattern Recognition with Fuzzy Objective Function Algorithms.

[33] Yousefi-Banaem H., Kermani S., Sarrafzadeh O., Khodadad D. An Improved Spatial FCM Algorithm for Cardiac Image Segmentation. Proceedings of the 13th Iranian Conference on Fuzzy Systems.

[34] Chuang K.S., Tzeng H.L., Chen S., Wu J., Chen T.J. (2006). Fuzzy C-means Clustering with Spatial Information for Image Segmentation. Comput. Med. Imaging Graph..

[35] Qiu X.P., Sun R.X., Yu D., Yang D. (2016). Method of Establishing Membership Functions of Car-following Behavior based on Fuzzy Clustering. Appl. Res. Comput..

[36] Lee J.S. (1980). Digital Image Enhancement and Noise Filtering by Use of Local Statistics. IEEE Trans. Pattern Anal. Mach. Intell..

[37] Frost V.S., Stiles J.A., Shanmugan K.S., Holtzman J.C. (1982). A model for radar images and its application to adaptive digital filtering of multiplicative noise. IEEE Trans. Pattern Anal. Mach. Intell..

[38] Kuan D.T., Sawchuk A.A., Strand T.C., Chavel P. (1985). Adaptive Noise Smoothing Filter for Images with Signal-Dependent Noise. IEEE Trans. Pattern Anal. Mach. Intell..

[39] Du P.J. (2002). Research on Filtering of RADARSAT Image. J. China Univ. Min. Technol..

[40] Wu B.K., Fan S.F. (2006). Evaluation of Performance of Deblurring of SAR Images based on Wavelet Transform. J. Hefei Univ. Technol..

[41] Simard M., Degrandi G., Thomson K.P.B., Benie G.B. (2002). Analysis of Speckle Noise Contribution on Wavelet Decomposition of SAR Images. IEEE Trans. Geosci. Remote Sens..

[42] Jarabo-Amores P., Rosa-Zurera M., Mata-Moya D.D.L., Vicen-Bueno R., Maldonado-Bascon S. (2011). Spatial-Range Mean-Shift Filtering and Segmentation Applied to SAR Images. IEEE Trans. Instrum. Meas..

[43] Asli O., Zuhal A. A Comparison of SAR Filtering Technioues on Agricultural Area Identification. Proceedings of the ASPRS 2010 Annual Conference.

[44] Ling F.L., Wang X.Q., Chen Y.Z. (2004). Evaluation and Study of Speckle Reduction Effect of SAR Images—Taking the Fujian Coastal Zone as an Example. Adv. Mar. Sci..

[45] Wang Y.H., Fan W.Y., Zhang J.H. (2015). The Comparison and Analysis of SAR Image Filtering Methods. For. Eng..

[46] Zhang T., Yang X.M., Hu S.S., Su F.Z. (2013). Extraction of Coastline in Aquaculture Coast from Multispectral Remote Sensing Images: Object-Based Region Growing Integrating Edge Detection. Remote Sens..

[47] Thieler E.R., Himmelstoss E.A., Zichichi J.L., Ergul A. (2009). The Digital Shoreline Analysis System (DSAS) Version 4.0—An ArcGIS Extension for Calculating Shoreline Change.

[48] Thieler E.R., Himmelstoss E.A., Zichichi J.L., Ergul A. (2017). Digital Shoreline Analysis System (DSAS) Version 4.0—An ArcGIS Extension for Calculating Shoreline Change (Version. 4.4, July 2017).

[49] Dolan R., Hayden B., Heywood J. (1978). New Photogrammetric Method for Determining Shoreline Erosion. Coast. Eng..

[50] Li X., Zhang L.P., Ji C.C., Liu H.Y., Huang Q.H. (2014). Spatiotemporal Changes of Jiangsu Coastline: A Remote Sensing and GIS Approach. Geogr. Res..

